# 2-years retrospective observational case-control study on survival and marginal bone loss of implants in patients with hereditary coagulopathies

**DOI:** 10.4317/medoral.25997

**Published:** 2023-04-26

**Authors:** Manuel Pérez-Fierro, Lizett Castellanos-Cosano, Juan Antonio Hueto-Madrid, Julián López-Jiménez, Ramiro José Núñez-Vázquez, Guillermo Machuca-Portillo

**Affiliations:** 1 Postgraduate lecturer. Department of Estomatology, University of Seville, Spain; 2 Professor. Department of Estomatology, University of Seville, Spain; 3 Chairman. Hospital Quirón Salud. Unidad de Cirugía Oral y Maxilofacial. Barcelona, Spain; 4 Chairman. Hospital NenDeu. Unidad de Odontology en Pacientes con Necesidades Especiales, Barcelona, Spain; 5 Chairman of the Haemophilia Unity, Haematology Service, Virgen del Rocío University Hospital, Sevilla, Spain; 6 Full Professor and Chairman. Department of Estomatology, University of Seville, Spain

## Abstract

**Background:**

Evaluating 2-years implant loss and marginal bone loss in patients with hereditary coagulopathies, comparing with a healthy control group.

**Material and Methods:**

37 implants in 13 patients (17 haemophilia A, 20 Von-Willebrand disease) versus 26 implants in 13 healthy patients. Data measured through Lagervall-Jansson index (after surgery, at prosthetic loading, at 2 years). Statistics: Chi-square, Haberman’s, ANOVA, Mann-Whitney-U. Significance *p*<0.05.

**Results:**

Haemorrhagic accidents in 2 coagulopathies patients (non-statistical differences). Hereditary coagulopathies patients suffered more hepatitis (*p*<0.05), HIV (*p*<0.05) and less previous periodontitis (*p*<0.01). Non-statistical differences in marginal bone loss among groups. 2 implants were lost in the hereditary coagulopathies and none in the control group (non-statistical differences). Hereditary coagulopathies patients had longer (*p*<0.001), and narrower implants (*p*<0.05) placed. 43.2% external prosthetic connection in hereditary coagulopathies patients (*p*<0.001); change of prosthetic platform more frequent in control group (*p*<0.05). 2 implants lost: external connection (*p*<0.05). Survival rate 96.8% (hereditary coagulopathies 94.6%, control group 100%).

**Conclusions:**

Implant and marginal bone loss at 2 years is similar in patients with hereditary coagulopathies and control group. Precautions should be taken on the treatment for hereditary coagulopathies patients, through prior haematological protocol. Implant loss only occurred in in a patient with Von-Willebrand´s disease.

** Key words:**Dental implants, inherited coagulation disorders, haemophilia A/B, Von Willebrand disease, marginal bone loss.

## Introduction

Hereditary coagulation disorders are considered “rare diseases” (the total prevalence of the disease being 7.7:100000). The main hereditary coagulation disorders known are haemophilia A and Von Willebrand disease, which make up 95-97% of coagulation pathologies (1-3).

Haemophilia A is a recessive hereditary coagulation disorder linked to the X chromosome due to a mutation in the F9 gene; however, in 30% of cases it is produced by a sporadic mutation. This mutation causes a deficiency of coagulation factor VIII (4,5). The severity of the disease will be related with the symptoms and are the same for all subtypes, consisting of spontaneous or traumatic bleeding, haemorrhages in the central nervous system (brain haemorrhages), muscle haematoma, haemarthrosis (70-80%) and haemophilic arthropathy (2,4,6). These haemorrhages may also appear at the level of the oral cavity in different locations, such as (from more to less common) the gum, labial frenulum and tongue, as these are areas with high capillarity. Ecchymosis and petechiae may also appear (7). With regard to treatment, factor concentrates derived from plasma are used, but 60% of patients were infected with the hepatitis C or HIV virus by this method.

Von Willebrand’s disease is an autosomal dominant disorder, which causes a reduction of the “Von Willebrand factor” plasma protein, or an alteration to it. This protein acts stabilising the factor VIII and making the interaction of platelets with the wall of blood vessels possible when there is vascular damage, as it circulates in a non-covalent complex along with the factor VIII. This pathology affects 0.8-2% of the general European and American population (2,7).

There is no long-term retrospective case-control study on the placement of implants in hereditary coagulopathies patients. Only one prospective observational study at 4 months has been published (8). Furthermore, no clinical trial has been published and there are only some isolated clinical cases. Therefore, the scientific evidence provided is minimal in terms of suggesting that the placement of implants may be the best treatment option for patients with hereditary coagulopathies. This study is proposed, with the following objectives: 1) evaluating the survival rate of implants and marginal bone loss 2 years after prosthetic loading in patients with hereditary coagulopathies (haemophilia A and Von-Willebrand disease) and comparing them with the implants placed in a control group of patients without coagulopathies; 2) evaluating the relationship between suffering an hereditary coagulopathy and complications in implant therapy.

## Material and Methods

Retrospective observational case-control study carried out under the guidance of the Faculty of Dentistry of the University of Seville (Spain). This study was approved by the human subject’s ethics board of the “Ethics Committee and of the Virgen Macarena-Virgen del Rocio University Hospitals” with the protocol number “peiba_DictamenFavorable2017112994246” and was conducted in accordance with the Helsinki Declaration of 1975, as revised in 2013. All the participants in the study signed a written informed consent.

In the study group, 39 panoramic radiographies were taken from 13 patients, and another 39 panoramic radiographies from 13 patients in the control group. The first panoramic radiography was taken immediately after placement of the implant, the second after 3 months, when undertaking the prosthetic loading, and the third 2 years after loading of the implant. The panoramic radiographies were evaluated for the marginal bone loss implanted following the radiological classification proposed by Lagervall and Jansson for diagnosis of marginal bone loss around implants (9).

- Study population

The sample group was made up of 13 patients, of which 6 had been diagnosed with haemophilia A and 7 with Von-Willebrand disease. The control group was made up of 13 patients without hereditary or acquired coagulopathies.

- Inclusion and exclusion criteria:

Criteria for inclusion of cases:

1. Patients of the Dental Practice of the Faculty of Dentistry of Seville (Spain), of Hospital Nen Deu and Hospital Quirón Salud in Barcelona (Spain), who had implants placed following the clinical protocol designed by the Dental Teaching Unit for Patients with Special Needs and the Hereditary Coagulopathies Unit of Hospital Universitario Virgen del Rocío in Seville (Spain) (10), and who had previously been diagnosed as having hereditary coagulopathies, either haemophilia A or Von-Willebrand disease.

2. Patients with accumulation of bacterial plaque on less than 10% of surfaces. The plaque index was classified using the O'Leary index (11).

1. Patients without periodontitis or active cavities at the time of selection.

Criteria for exclusion of cases:

2. Patients whose implants were not placed at the centres indicated above.

3. Patients without haemophilia A or Von-Willebrand disease (only for the group of cases).

4. Patients who receive treatment which may potentially affect bone metabolism, such as long-term steroids, bisphosphonates or monoclonal antibodies.

5. Patients treated with short implants.

6. Immediately loaded implants.

7. Patients with active or untreated periodontal disease.

8. Patients who smoke.

Criteria for inclusion of controls:

1. Patients without systemic pathologies, or with treated mild systemic pathology, which will not directly affect coagulation with implants placed in the same period of time.

2. Patients with a similar age range to that of the control group.

3. Patients with accumulation of bacterial plaque on less than 10% of surfaces. The plaque index was classified using the O'Leary index (11).

4. Patients without periodontitis or active cavities at the time of selection.

Criteria for exclusion of controls:

1. Patients whose implants were not placed at the centres indicated above.

2. Patients with haemophilia A or Von-Willebrand disease (only for the control group).

3. Patients who receive treatment with antiplatelet and anticoagulation drugs.

4. Patients who receive treatment which may potentially affect bone metabolism, such as long-term steroids, bisphosphonates or monoclonal antibodies.

5. Patients treated with short implants.

6. Immediately loaded implants.

7. Patients with active or untreated periodontal disease.

8. Patients who smoke.

9. Patients with untreated and controlled serious illnesses which may affect osseointegration (cardiovascular, respiratory, endocrine, etc.).

10. Patients who have suffered HIV or hepatitis C.

To take into account the clustering effects, one implant was selected per patient in accordance with the following random procedure:

1. Generating a random number between 0 and 1 for each patient.

2. Determining which implant is selected for each patient based on the random number given and the total number of implants that will be placed in the patient.

3. Allocation of a sequential number to each implant in each patient.

4. Identification of the implant chosen in the second procedure.

5. Selection of a sample with the implants chosen.

After the previous procedure, 26 implants were selected: there were 13 implants in the study group and 13 in the control group. To evaluate the variability of results, the statistical procedure used in the implant sample was also applied to these implants.

- Radiological study

A radiological study was carried out through panoramic radiographies to examine the marginal bone loss of the peri-implant area in the 13 cases and 13 controls. The method used was that described by Lagervall and Jansson (9) and validated for use in this type of study by Corcuera-Flores *et al*. (12). This method divides implants into four groups based on their marginal bone loss:

Level 0: implants without marginal bone loss.

Level 1: marginal bone loss of one third or less than one third of the total length of the implant.

Level 2: marginal bone loss greater than one third but less than two thirds of the total length of the implant.

Level 3: marginal bone loss greater than two thirds of the total length of the implant.

Level 4: A fifth group was added for patients who lost the implant for statistical analysis (12).

The demographic and clinical variables were taken from the medical histories of the patients and the essential data was verified.

Panoramic radiographies taken at the time of placement of the implants were compared with those taken after 3 months, at the time of loading the implants, and with those taken two years after prosthetic loading, to verify that the bone defects around the implants were mainly due to peri-implantitis and not a defect caused during placement. All panoramic radiographies were checked by the same examiner. The panoramic images were acquired with a Planmeca Pro Max®. The increase of panoramic radiographies was 1-1.

- Haematological protocol

For all patients with hereditary coagulopathies, the following haematological protocol was carried out:

a) Patients with haemophilia A:

Prior to the procedure, patients with serious/moderate haemophilia A were administered factor concentrates to increase the level of factor VIII between 60% and 80%. In patients with mild haemophilia (>10%) desmopressin iv was used with a dose of 0.3g/kg in weight.

b) Patients with Von-Willebrand disease:

In patients with type 1 Von-Willebrand disease, desmopressin was used in the same doses as in haemophilia A and factor VIII/ factor Von-Willebrand concentrates in patients with type 2 and 3 Von-Willebrand disease.

Antifibrinolytics were administered to all patients during a period of between 5 and 10 days (1g tranexamic acid pills every 6 hours from the night before the surgical procedure). All patients were made to bite a gauze soaked in tranexamic acid for 30 minutes and were prescribed antibiotics (amoxicillin 875mg with clavulanic acid 125mg). They were also prescribed analgesics, mainly paracetamol in doses of 500mg every 4-8 hours, depending on the case (10,13).

- Characteristics of the implants and prostheses:

The implants placed were made of sandblasted, acid etched grade IV titanium, with lengths of 8, 10, 11.5, 12, 13, and 15mm; short implants were not used in this study. To facilitate data analysis, the lengths of the implants were grouped into the following categories: (“from 8-12mm” and “over 12mm”). The widths of the implants were 3.3, 3.4, 3.75, 4.0, 4.1, 4.2, 4.25 and 4.8mm. In the same sense, to facilitate data analysis, the widths of the implants were grouped into the following categories (“under 3.75mm”, “from 3.75 to 4.25mm” and “over 4.25mm”). The implants placed were of the following implant systems: Microdent M-4212-SP®, which had an external connection, were self-tapping with sandblasted helical knurls which reached the smooth neck and were subjected to an abrasive surface treatment of extreme cleaning, to give the surface a microrough surface; Straumann SP® with an internal connection, this implant having a solid cylindrical threaded body with a thread design which includes an angle of 15º and a thread pitch of 1.25mm, with a smooth transgingival neck of 2.8mm; Straumann BL®, with an internal connection, cylindrical, with a straight neck at the bone level and a thread pitch of 0.8mm; Biomet-3i-Osseotite®, which had an external connection, were self-tapping with sandblasted helical knurls which reached the smooth neck without microroughness on the neck; Ticare Osseus® with an external hexagon and machined smooth neck of 1.5mm; and Ticare Inhex® with an internal connection, self-tapping, with microthread on the coronal area and change of platform. All implants and prostheses were made scrupulously following the instructions of each manufacturer.

With regard to facilitating data analysis, all implants were grouped into the following categories:

1. Position of the implant with regard to the bone crest: “At bone level” or “At tissue level”

2. Implant-pillar connection: “Internal” or “External”

3. Change of prosthetic platform: “Yes” or “No”.

All prostheses were screwed, both single crowns and 3-unit FDPs, and even overdentures (any removable dental prosthesis which covers and rests on dental implants, also known as overlay prosthesis or superimposed prosthesis). No pillar was cemented.

To avoid bias, all measurements were carried out by the same calibrated examiner. Each radiograph was assigned a random number so that the examiner could not know which group of patients they belonged to. This number was only known to the senior examiner and revealed at the end of the investigation.

- Statistical analysis

All variables were subjected to a descriptive analysis. Normality between the numerical variables was determined applying the Kolmogorov-Smirnov test. Any interaction between the qualitative variables was evaluated through the chi-squared test and Haberman’s post-hoc test, which allowed all researchers to calculate the importance of each subvariable independently. The interactions between the categorical and numerical variables were evaluated applying the ANOVA test for variables with normal distribution and the Mann-Whitney U test for those which did not have a normal distribution.

The survival rate was evaluated as the percentage of implants which continued to function after 2 years, in the total sample, and in each study group.

The statistical analysis was carried out with SPSS Version 25 for Windows 7.

The statistical significance level was *p*<0.05.

## Results

The average age of patients at the time of placement of implants was 57.73±15.51 years (55.69 for the hereditary coagulopathies group and 59.76 for the control group). In the study group, 37 implants were placed in 13 patients (17 in haemophilia A, 20 with Von-Willebrand disease), of which 20 implants were placed in the upper maxilla and 17 in the lower maxilla. In the control group, 26 implants were placed in 13 healthy patients, 10 in the upper maxilla and 16 in the lower maxilla. 16 implants were placed in the anterior region of the mouth (between the canines) and 47 in the posterior region (molars and premolars). Within the study group, 9 implants were placed in the anterior region and 28 in the posterior region: in the control group, 7 in the anterior region and 19 in the posterior region. There were no significant differences between the study groups with regard to the location of the implants. All details of the implants placed, the prosthetic load and the characteristics of the patients studied are shown in Table 1.

When one implant was selected per patient, the size of the sample was reduced to 26 implants.

When the levels of marginal bone loss of Lagervall and Jansson (9) were analysed 3 months after placement, at the time of the prosthetic loading, only 1 (2.7%) implant of the study group and 1 (3.8%) of the control group fell within Level 0 (no marginal bone loss). The 36 (97.3%) implants remaining within the study group with coagulopathies and the 25 (96.2%) within the control group fell within Level 1 (1/3 or less marginal bone loss). When one implant was evaluated per patient, no implants [0] in the study group and 1 (7.7%) in the control group fell within Level 0 (no marginal bone loss), while 13 (100%) in the study group and 12 (92.3%) in the control group fell within Level 1 (1/3 or less marginal bone loss). No statistically significant differences were found between the groups.

Table 2 shows the survival rates and marginal bone loss levels of Lagervall and Jansson (9) in comparison with the study and control groups 2 years after the prosthetic loading. No significant differences were found between the groups when comparing all implants, or when evaluating one implant per patient. When the 63 implants were evaluated, 2 (12.5%) implants were lost, and none [0] with external connection (χ2 *p*<0.05); likewise, the 2 (14.3%) implants lost were located in the posterior region of the maxilla and none [0] in the anterior maxilla or posterior mandibular region (χ2 *p*<0.05). When one implant was analysed per patient (*n*=23), differences were established with regard to the length of the implant, with 1 (25.0%) implant of over 12mm being lost, and none of 8-12mm (χ2 *p*<0.05). Other variables such as the type of coagulopathy, having previously suffered successfully treated periodontitis, the width of the implant, the positioning of the implant with regard to the bone crest, or having a change of prosthetic platform were not related with the loss of the implants, whether the whole sample (*n*=63) or the cluster (*n*=26) were studied.

The relationships that the characteristics of the implant and the type of prosthetic load may have on the marginal bone loss or loss of implants were evaluated, comparing the two study groups. When the 63 implants placed were considered, the group of patients with coagulopathies had had implants placed with a smaller width, of 3.86±0.31mm, compared with the control group, which were 4.11±0.45mm (Mann-Whitney U *p*<0.05). Conversely, in the study group, implants of a greater length were placed, of 11.35±2.09mm, while in the control group they were 9.54±1.53mm (Mann-Whitney U *p*<0.001). When one implant per patient was considered (26 randomly selected implants), no significant differences were established between the group of patients with coagulopathies compared with the control group (Mann-Whitney U N.S.), with regard to MBL or implant loss. Conversely, in the study group, implants of a greater length were placed, of 11.65±1.89mm, while in the control group they were 10.0±1.41mm (Mann-Whitney U *p*<0.05). In Table 3, all the different characteristics of the implants and their prosthetic loads referring to the study groups are shown.

**Table 1:**
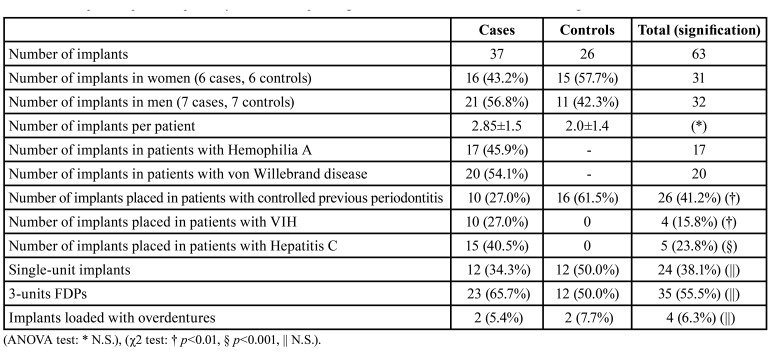
Description of placed implants by number of implants, gender, health condition and kind of loading.

**Table 2:**
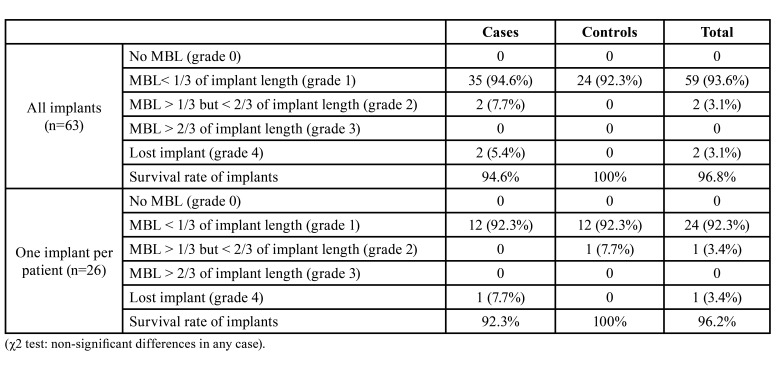
Marginal bone loss (MBL) comparing cases and controls two years after prosthetic loading.

**Table 3:**
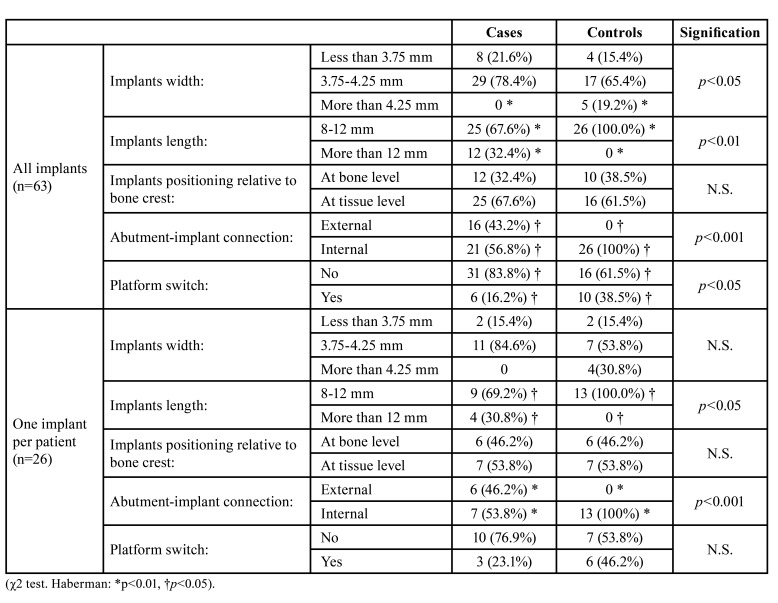
Description of the type of implants and the characteristics of the prosthodontic restorations according to the study group.

## Discussion

Patients with hereditary coagulopathies present a great aversion to surgical treatments, which limits their requesting the placement of implants. Additionally, technical difficulties for their placement lead dentists to not consider this type of treatment an option (7). 60% of these patients were contaminated years ago with transfusions infected with HIV and the hepatitis C virus. This has meant that the placement of implants has not been considered appropriate for some time. As a result, only isolated clinical cases have been published, which did not usually provide validaTable clinical protocols (14).

Before oral surgical procedures it is recommended to increase the deficient factor by 50%, or administer vasopressin, and use an antifibrinolytic locally along with other local measures (7,15,16). In fact, patients who take factor for prevention seem to have less risk of bleeding in oral surgery interventions, although regenerative surgeries for the increase of bone available are considered contraindicated for these patients (15). All surgical measures described has been respected in this study, have been undertaken on an outpatient basis, were always supervised by a specialised haematology service and may be responsible for the reduced rate of failure in the hereditary coagulopathies group, without statistical differences with the control group.

In these treatments, diagnosis is recommended through three dimensional images (computed tomography), in order to avoid the risk of inadvertently perforating corticals (especially the lingual cortical), which may cause severe bleeding in patients with coagulopathies (15). Thus, the pre-surgical diagnosis of this study was carried out, although to be able to work with a validated protocol, all measurements were carried out on orthopantomographs (9). No significant differences were found when comparing the marginal bone loss after 2 years between the study group and the control group, according to the criteria established by Lagervall and Jansson (9). Although the measurement in millimetres would provide greater precision and greater statistical power, the complexity of measuring the marginal bone loss in panoramic radiographies may impede precise measurements. For this reason, Lagervall and Jansson (9) proposed a scale for measuring marginal bone loss, which was selected for use in this study because it had been validated in similar previous publications (12), and may be of interest for comparing these results with those of other researchers.

The parameters related with marginal bone loss and implant loss have been measured from a second radiography taken at the time of prosthetic loading of implants, 3 months after their placement. If the radiographies had been taken one year after implantation, there would have been more time for remodelling the corresponding bone, and the comparisons of marginal bone loss with radiographies after 2 years may be more precise. Being a retrospective observational study, the radiographic material existing was limited to the radiographies taken at the time of the surgery, and 3 months later, when the prosthesis was loaded. It is noTable that in this study, in the measurements taken after 2 years, 94.6% of implants of the hereditary coagulopathies group had less than 1/3 marginal bone loss (Level 1), similar to that of the healthy control group (92.3%), in both cases similar to the average (93.6%) and to what has been described in other studies on healthy patients (17).

Studies on the placement of implants in patients with hereditary coagulopathies are so limited that only isolated clinical cases or extremely short series limited in time have previously been published. These studies generally reveal the success of implant therapy on a single patient. The scientific evidence is scarce and of a low level, as a total of only 6 articles published over the course of 22 years can be found (10,18-22), all of these communicating the results of a single clinical case and taking into account that based on the clinical images published, the case of Gornitsky *et al*. (19) may be the same as that of Rosen & Gornitsky (18), with different scientific considerations. Only one recent observational prospective study was observed, at 4 months on 10 patients (21 implants) with haemophilia A in selected patients (8). This retrospective case-control study at 2 years would be the only one with these characteristics published to date.

Despite the fact that hereditary coagulopathies may increase the risk of haemorrhage during the placement of implants, there is not yet certainty that they entail a contraindication for the success of implants, therefore failing due to this type of pathology. It has been published that it is preferable to avoid treatments consisting of maxillary sinus lifts and bone grafts, as these surgical therapies considerably increase the risk of haemorrhage (15,23). Nevertheless, some clinical studies published have used these surgical procedures on patients with Haemophilia B and Haemophilia A without postoperative complications observed during the monitoring period of the cases (15,23). Neither in this study, nor in the series of 21 implants in 10 patients published by Gatti *et al*. (8) were these techniques used on patients with hereditary coagulopathies.

In its study group, this retrospective observational study includes patients who had been infected with HIV and/or the hepatitis C virus. The presence of these concomitant diseases may complicate the postoperative period of the patient, being another of the factors to take into account in implant surgery on patients with hereditary coagulopathies. Castellanos-Cosano *et al*. (10), published a case on a patient with severe haemophilia A, HIV and hepatitis C, in which the edentulous sectors of the lower jaw were successfully rehabilitated through prosthesis over implants. In this study, being HIV or hepatitis C positive did not affect marginal bone loss or implant loss in any way, in the same way as in other published series, in which implants placed in patients with HIV had a survival rate of 98.3% after 6 years (14). Although the results of the study may be distorted due to the effect of these serious infections, the results obtained reinforce the clinical significance of these results, in an environment closer to the clinical reality of these patients.

Despite the haemostatic protocol used, in one case Von-Willebrand disease caused a haematoma during the placement of 2 implants, which were precisely those which were lost, after being loaded, during the course of the 2 years between the second radiographic test and the third. Control of the haemorrhage is considered of great interest, not only due to its importance for the health of the patient, but also for the long-term viability of the implants. In the protocol developed in this study, a systemic and local antifibrinolytic therapy was used. Further study is required to be able to draw conclusions on its level of efficacy in these surgeries (24). In a prior study by Franchini *et al*. (25) patients with type 1 Von Willebrand disease were treated with implants, being administered desmopressin 0.3mg/kg 1 hour before the intervention, achieving 30-50% of the normal factor values. Postoperative bleeding only occurred in one patient (14%), a similar situation to that of this study. Therefore, in patients with type 1 Von Willebrand disease, it appears safe to place dental implants after a treatment with desmopressin. For more severe illnesses, or in case of contraindication of desmopressin, transfusion of platelets may be necessary (26).

In the only series published, Gatti *et al*. (8) describe 3 cases of postoperative haemorrhage, which were controlled by the protocol described in this study, while in this study only 1 patient suffered this accident. In the aforementioned study (8), designed for 4 months, no implant loss is described, as occurred in this study during a similar period, causing the later loss of implants affected by the postoperative haematoma. Once again, the monitoring of these patients over the years is important, because although the survival rate of this study at 3 months was 100%, a survival rate of 94.6% was revealed in the group with hereditary coagulopathies after 2 years of prosthetic load, which is slightly lower than that published by Jamcoski *et al*. (17) for patients without treated coagulopathies with implants with hydrophobic surfaces (97.87%), in which the monitoring period was much longer (5 years) than the period of this study. In any case, due to the limited number of cases, and the fact that the only 2 implants lost were from the same patient, until longer-term studies are carried out, more specific conclusions should not be drawn.

Although in this study patients with hereditary coagulopathies had longer and narrower implants placed than those in the control group, it does not appear that these circumstances worked in favour of or against the loss of the implants. It is noTable that the cases used the external prosthetic connection in a significant number of restorations, as the 2 implants lost in the patient affected by Von-Willebrand disease had an external prosthetic connection. Although there are studies that demonstrate the advantages of an internal connection compared with the external connection, above all if it is through a Morse taper (27), due to the limitations of the sample, it is not thought that this study could be conclusive with regard to the selection of one connection or another in patients with hereditary coagulopathies.

It can be concluded that both marginal bone loss and the survival rate of implants after 2 years are similar in patients with hereditary coagulopathies and in patients of the healthy control group. Patients with hereditary coagulopathies (Von-Willebrand disease) are the only ones who lost implants later and in relation with a haemorrhagic process, although marginal bone loss after 2 years was similar to the control group. Therefore, strict compliance with the established haematological protocols is necessary. Further study with more clinical cases and longer monitoring periods are necessary; until then, implants may be placed in hereditary coagulopathies patients (haemophilia A and Von-Willebrand disease). Under the same clinical conditions, the results may be similar to those obtained with healthy patients. However, dentists should be very meticulous when opting for rehabilitation with implants in patients with hereditary coagulopathies, and always carry out a prior consultation with the haematology services to establish the action protocols.
